# Two types of bone necrosis in the Middle Triassic *Pistosaurus longaevus* bones: the results of integrated studies

**DOI:** 10.1098/rsos.170204

**Published:** 2017-07-26

**Authors:** Dawid Surmik, Bruce M. Rothschild, Mateusz Dulski, Katarzyna Janiszewska

**Affiliations:** 1 Park of Science & Human Evolution, 1 Maja 10, 46-040 Krasiejów, Poland; 2 Faculty of Earth Science, University of Silesia, Będzińska 60, 41-200 Sosnowiec, Poland; 3 Carnegie Museum, 4400 Forbes Ave, Pittsburgh, PA 15213, USA; 4 West Virginia University School of Medicine, Morgantown, WV 26506, USA; 5 Silesian Centre for Education and Interdisciplinary Research, 75 Pułku Piechoty 1A, 41-500 Chorzów, Poland; 6 Institute of Material Science, University of Silesia, 75 Pułku Piechoty 1A, 41-500 Chorzów, Poland; 7 Institute of Paleobiology, Polish Academy of Sciences, Twarda 51/55, 00-818 Warsaw, Poland

**Keywords:** bone necrosis, decompression syndrome, septic arthritis, Triassic, paleopathology

## Abstract

Avascular necrosis, diagnosed on the basis of either a specific pathological modification of the articular surfaces of bone or its radiologic appearance in vertebral centra, has been recognized in many Mesozoic marine reptiles as well as in present-day marine mammals. Its presence in the zoological and paleontologic record is usually associated with decompression syndrome, a disease that affects secondarily aquatic vertebrates that could dive. Bone necrosis can also be caused by infectious processes, but it differs in appearance from decompression syndrome-associated aseptic necrosis. Herein, we report evidence of septic necrosis in the proximal articular surface of the femur of a marine reptile, *Pistosaurus longaevus*, from the Middle Triassic of Poland and Germany. This is the oldest recognition of septic necrosis associated with septic arthritis in the fossil record so far, and the mineralogical composition of pathologically altered bone is described herein in detail. The occurrence of septic necrosis is contrasted with decompression syndrome-associated avascular necrosis, also described in *Pistosaurus longaevus* bone from Middle Triassic of Germany.

## 1. Introduction

Pistosaurs (Pistosauridae) are marine reptiles, considered as a transitional form between Triassic stem-sauropterygians, which inhabited near shores, and advanced, open marine Jurassic and Cretaceous plesiosaurs. Pistosaurid remains have been found in Europe, North America and China [1–10], documenting their distribution in the Triassic world on both sides of the Pangea supercontinent. Pistosaurs are considered a sister taxon of plesiosaurs on the basis of anatomical features, including the structure of the pectoral and pelvic girdles (compare in [4]). Moreover, histology of long bones (radially vascularized fibro-lamellar bone) suggests fast growth and tolerance for cold temperatures [11,12], which is also shared with post-Triassic plesiosaurs [12].

Decompression syndrome (DCS), known also as Caisson's disease or ‘the bends’ [13], affects a body exposed to rapidly diminishing external pressure related to rapid ascent in the water column. DCS causes necrosis of bone (referred to as avascular necrosis, AVN), manifesting macroscopically as bone infarction and subsidence of the proximal articular surfaces of humeri and femora. Such subsidence is the direct evidence of decompression syndrome [14]. It has been identified in Mesozoic marine reptiles—sea turtles, mosasaurs and ichthyosaurs [14–18], as well as sauropterygians [19]. Avascular necrosis is common in post-Triassic sauropterygians, indicating that they were susceptible to decompression syndrome because of prolonged and repetitive diving behaviour in these marine reptiles.

Decompression syndrome-associated bone necrosis is also called aseptic necrosis, to distinguish it from another form of AVN, septic necrosis. The latter is caused by an infectious process referred to as septic arthritis. It usually resulted in abnormal new bone formation with cauliflower-like appearance [20] and characteristic filigree texture. It is known in living tetrapods but has only been identified in fossil records to date in Cretaceous duck-billed dinosaur [20].

Herein we present two of the mentioned types of bone necrosis, recognized in *Pistosaurus longaevus* limb bones. A classic bends-related AVN was present in a humerus, and a partially preserved pistosaur femur was investigated in detail, showing evidence of infection type of bone necrosis. We applied high-resolution X-ray microcomputed tomography (XMT) to demonstrate infection-mediated abnormal bone formation (herein referred to plaque) and joint surface collapse due to decompression syndrome. Moreover, we have examined the chemical composition of the necrotic plaque and non-altered bone, showing differences resulting from new bone tissue formation in the pathological conditions.

## 2. Material and methods

### 2.1. Institutional abbreviations

SUT, Museum of Geology, Silesian University of Technology (Gliwice, Poland); NME, NaturkundeMuseum Erfurt (Erfurt, Germany); SMF, Naturmuseum Senckenberg (Frankfurt, Germany); MHI, Muschelkalk Museum Hagdorn Ingelfingen (Ingelfingen, Germany); GIUS, Department of Paleontology, Faculty of Earth Science, University of Silesia (Sosnowiec, Poland).

### 2.2. Literature records

The unusual appearance of proximal joint surfaces (including focal subsidences and bone enlargements) captured the interest of the present authors (DS, BMR) on the detailed drawings of pistosaur long bones from Bayreuth in H. Meyer's treatise on Middle Triassic reptiles [21, plate 49]. Unfortunately, these specimens are no longer available in public repositories. This observation prompted us to investigate three limb bones from Bindlach near Bayreuth (Bavaria, Germany) and Bad Sulza (Thuringia, Germany) previously illustrated by C. Diedrich [4, figs 7C–F, 16B].

### 2.3. The specimens

The proximal part of the femur (specimen no. SUT-MG/F/Tvert/43-1) studied here in detail came from the Boruszowice Formation of Rybna, district of Tarnowskie Góry town, located in Upper Silesia, southern Poland. Its designation as *Pistosaurus longaevus* is based on personal observation (DS), referencing comparative bone material from the Museum of Natural History, Berlin, Germany. Moreover, three other pistosaur long bones (MHI 931, NME 78.341 and SMF R 2011) from Germany were studied macroscopically. As reference samples in spectral studies, we presented carbonate host rock surrounding specimen SUT-MG/F/Tvert/43-1 (herein referred to host rock) and the femur of an extant Galápagos marine iguana (*Amblyrhynchus cristatus*, GIUS-12-3628). The extant bone tissue used in our study was collected as an isolated bone with the permission of the appropriate local authorities for research purposes (see Ethics).

### 2.4. Stratigraphy

The historical pistosaur findings from Tarnowskie Góry area (Upper Silesia) come from the Wilkowice and Boruszowice formations (Illyrian/Fassanian), similar in age to that of the German localities. According to Szulc [22], the Boruszowice Formation is considered to be isochronous to the Meissner Formation and correlates with the Bindlach and Hegnabrunn formations, from where specimens MHI 931, NME 78.341 and SMF R 2011 come (compare [4,5]). Numerous pistosaur remains from the Silesian Upper Muschelkalk (Middle Triassic) are housed in the Museum of Natural History in Berlin, Germany. These remains were studied by one of us (DS) in 2012. According to the labels, these specimens come from several historical localities of Rybna, Tarnowskie Góry (Tarnowitz), Opatowice (Opatowitz) and Laryszów (Larischof), the locations where only the Wilkowice Formation and Boruszowice Formation limestones are exposed. Several dozen pistosaur remains (mostly vertebrae) are housed in the Museum of Geology, Silesian University of Technology (Gliwice, Poland), including the specimen SUT-MG/F/Tvert/43-1 investigated here.

### 2.5. Acid treatment

The specimen SUT-MG/F/Tvert/43-1 was treated with 99.9% pure, non-buffered acetic acid (Avantor Performance Materials Poland S.A.; POCH Polish Chemicals Reagents, Gliwice, Poland; CAS identification number 64-19-7; WE identification number 200-580-7), diluted in demineralized water to a concentration not more than 10%. The proximal epiphyseal part of the specimen was submerged (see electronic supplementary material, figure S1) in acetic acid solution to remove limestone sediment (host rock residuum), subsequently rinsed in demineralized water and dried in a desiccator with moisture absorbing silica gel.

### 2.6. Raman spectroscopy

A WITec confocal Raman microscope CRM alpha 300 equipped with solid-state laser (*λ* = 532 nm) and a CCD camera (Laboratory of Raman Spectroscopy in Silesian Centre for Education and Interdisciplinary Research, Chorzów, Poland) were applied to determine the degree of bioapatite crystallinity and, indirectly, the chemical composition through analysis of phosphate and carbonate groups. An air Olympus MPLAN (50×/0.76NA) objective and monochromator with a 600 line mm^−1^ grating were used. All spectra were accumulated by 20 scans with an integration time of 120 s and a resolution of 3 cm^−1^. The spectrometer's monochromator was calibrated using the Raman scattering line of a silicon plate (520.7 cm^−1^). The fluorescence and baseline correction, as well as peak fitting analysis by Voigt function, were performed using the GRAMS software package.

### 2.7. Fourier transform infrared spectroscopy (FTIR)

Agilent Cary 640 FTIR spectrometer equipped with a standard source and a DTGS Peltier-cooled detector were used to follow the H-bonding pattern as well as the impact of hydroxide and molecular water incorporation on the modification of crystal structure of bone apatite. Surface water absorption was analysed in the referenced sample. The spectra were collected using a GladiATR diamond accessory (Pike Technologies) in the 4000–400 cm^−1^ range, with a spectral resolution of 4 cm^−1^ and accumulating 16 scans. The baseline correction was done and water vapour and carbon dioxide were subtracted from each spectrum. Peak fitting analysis was carried out with the Voigt function in the GRAMS software package.

### 2.8. Computed tomography

The initial computed tomography (CT) studies of specimen SUT-MG/F/Tvert/43-1 were performed with a GE Healthcare Discovery CT750 HD 64-channel computed X-ray tomograph unit (Department of Diagnostic Imaging of Regional Hospital of Trauma Surgery, Piekary Śląskie, Poland). The sample was exposed at 44.07 mGy, at 640 mA. CT scans were recorded as DICOM image files and processed and analysed using the GE Healthcare AW VolumeShare software. The dataset of initial CT scanning is not shown in the paper.

### 2.9. X-ray microcomputed tomography

The more detailed microtomographic data of specimen SUT-MG/F/Tvert/43-1 were collected with an XRadia MicroXCT-200 imaging system equipped with a 90 kV/8 W tungsten X-ray source in the Laboratory of Microtomography, Institute of Paleobiology, Polish Academy of Sciences, Warsaw. The scans were performed using the following parameters: voltage, 80 kV; power, 8 W; exposure time, 45 s; voxel size, 46.11 µm. Radial projections were reconstructed with the XMReconstructor software (8-section low contrast ring removal was used to reduce ring artefacts). For 3D imaging of bone, serial XMT sections were obtained with Avizo 7.0 Fire Edition software.

## 3. Results

### 3.1. Specimen SMF R.2011

**Figure 1.**
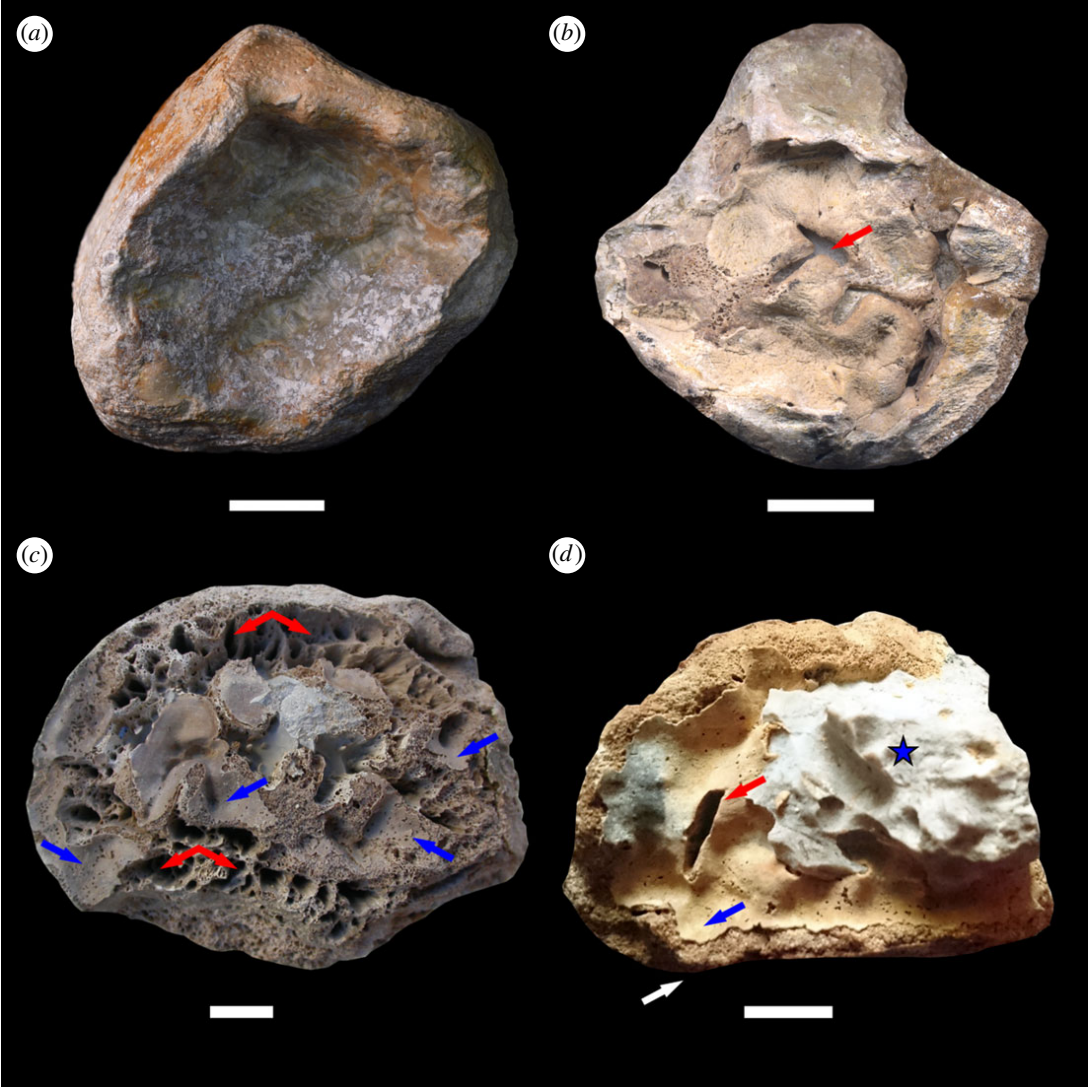
Pistosaur limb bone from Germany and Poland, visible from the proximal joint views. (*a*) The specimen SMF R.2011 presents typical joint surface collapse (subsidence) as a result of bends-related AVN. (*b*) The specimen NME 78.341 shows infectious process-related filigree texture and irregular disruption (cauliflower-like form) of pathologically overgrown bone tissue with draining sinus identified (red arrow). (*c*) The specimen MHI 931 shows significant degradation of the articular surface as a result of infectious process, numerous draining sinuses (several pointed by red arrows) and islets of pathological plaque (blue arrows). (*d*) Specimen SUT-MG/F/Tvert/43-1 shows both a collapsed articular surface and filigree texture (plaque) with draining sinus (red arrow). Note the presence of a thin plaque of bone formation (blue arrow) superimposed on normal, non-altered bone tissue (white arrow). Blue asterisk indicates host rock (limestone) covering the surface of the specimen. Blue and white arrows and blue asterisk indicate the area of samples for Raman and infrared analyses. All scale bars equal 10 mm.

An isolated, complete humerus (figure 1*a*) from Bindlach, ascribed to *Pistosaurus longaevus*, with collapse (subsidence) of the articular surface defect of the type seen with avascular necrosis from bends was examined. The margins are continuous, surrounding a depressed articular surface, which has collapsed onto underlying bone.

### 3.2. Specimen NME 78.341

A complete femur (figure 1*b*) from Bad Sulza is characterized with irregular, discontinuous margins surrounding a collapsed area with irregular base. Irregular disruption of that base with new bone formation and a draining sinus as well as cauliflower-like appearance is characteristic of an infectious process (see also electronic supplementary material, figure S2).

### 3.3. Specimen MHI 931

The complete femur (figure 1*c*) is characterized with extremely altered joint surface at the proximal end with numerous draining sinuses and islets of thin, amorphous pathological plaque (figure 1*c* and see electronic supplementary material, figure S3).

### 3.4. Specimen SUT-MG/F/Tvert/43-1

**Figure 2.**
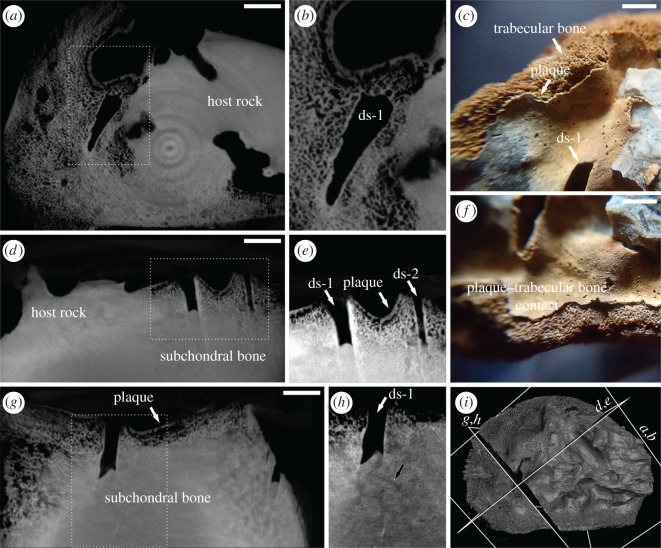
XMT sections and macro-photographic images of specimen SUT-MG/F/Tvert/43-1. (*a*) Transverse XMT section of joint surface showing the trabecular texture of the bone-like pathological plaque with (*b*) enlarged area showing details of necrotic plaque and the largest draining sinus (ds-1). (*c*) and (*f*) macro-photographic images showing details of surface of proximal head of femur with the visible largest draining sinus (ds-1) and continuous contact between pathological plaque and trabecular bone; (*d*), enlarged in (*e*), XMT section showing the superficial necrotic plaque and two draining sinuses (ds-1, ds-2); (*g*) XMT section showing the pathological plaque, enlarged in (*h*), black arrow shows suspected vascular canals; (*i*) 3D visualization of joint surface with section lines corresponding to planes presented on *a* to *h*. All scale bars equal 5 mm.

This is the proximal part of a pistosaur femur, 80 mm long, 56 mm in the widest section with focal depression of the proximal articular surface (figure 1*d*). The host rock covering the region of interest (figures 1*d*, and 2*a*) was partially removed by chemical dissolution (acetic acid) instead of mechanical preparation to avoid damage of the fragile, pathologically affected area. The focal depression manifests as a thin plaque with rare (in contrast with subchondral bone) nutritional foramina, appearing as black spots (figures 1*d* and 2*c*,*f*) and at least two split-like draining sinuses on the surface of the dead bone (figure 1*d*, red arrow). The surface is characterized by a filigree periosteal reaction (figure 1*d*, blue arrow), documenting the infectious origin of the pathology. The detailed XMT scans of SUT-MG/F/Tvert/43-1, of the pathologically altered joint surface and the opposite (distal) side of the same specimen, reveal details of the internal bone structure (electronic supplementary material, figure S4). Channels can be distinguished within bone tissue, manifest as darker smudges (figure 2*h*, black arrows). These intraosseous channels seem to contact directly with the bottom of one draining sinus (figure 2*h*, black arrow).

### 3.5. The results of chemical studies on SUT-MG/F/Tvert/43-1 and control samples

**Figure 3.**
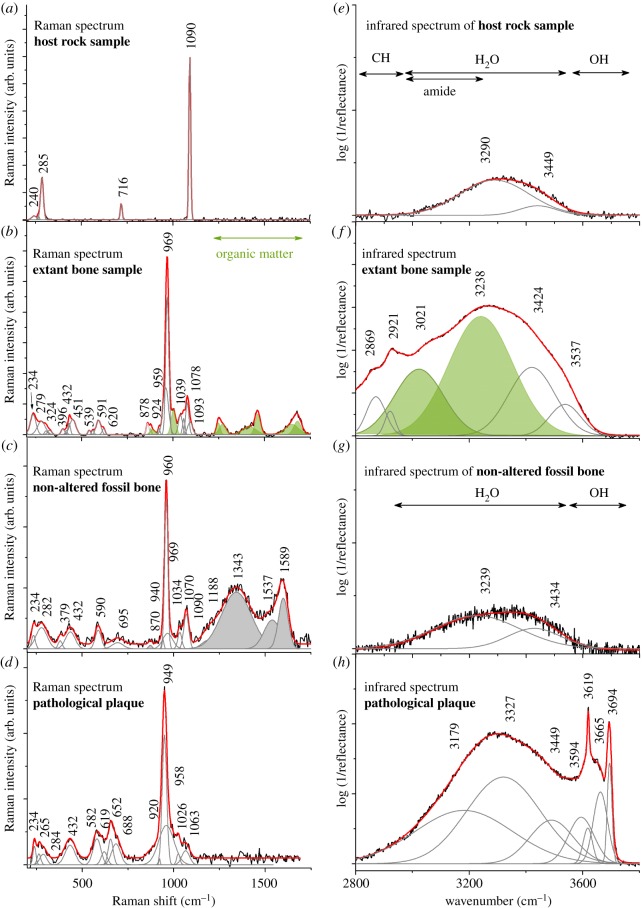
Raman and infrared spectra of the investigated samples. (*a*) Raman spectrum and (*e*) infrared spectrum of host rock sample covering specimen SUT-MG/F/Tvert/43-1 (compare figure 1*d*, blue asterisk). (*b*) Raman spectrum and (*f*) infrared spectrum of extant marine iguana bone sample (GIUS-12-3628). (*c*) Raman spectrum and (*g*) infrared spectrum of non-altered bone tissue of sample SUT-MG/F/Tvert/43-1. (*d*) Raman spectrum and (*h*) infrared spectrum of pathological plaque of sample SUT-MG/F/Tvert/43-1. Filled in green bands on Raman and infrared spectra are ascribed to organic matter. Grey bands on Raman spectrum are ascribed to carbonate units. Note that in extant bone sample, the carbonate bands might be overlapped by amide signal.

Pure acetic acid and limestone host rock samples were examined to rule out input of chemical treatment or host rock-derived carbonate substitutions on the result of spectroscopic studies of analysed samples (figure 3 for comparison). It is crucial to note that acetic acid provides surface protonation of calcium phosphates or replacement of calcium and/or hydroxide by protons [23]. The dissolution process is usually considered in terms of surface changes, wherein bulk modifications such as appearance of dislocations and structure alteration are negligible [24]. Hence, in SUT-MG/F/Tvert/43-1 samples (host rock, pathological plaque and non-altered cortical bone), no acetic acid remains and additional bands on FTIR or Raman spectra were found. Both spectroscopies methods focus at a depth of several micrometres. Changes in sample microstructure due to acetic acid treatment affected only the most superficial bone and do not compromise such techniques.

A detailed Raman analysis was performed to characterize the difference between the chemical composition and crystal structure of the two different areas of bone—pathological plaque (figure 1*d*, blue arrow) and non-altered bone (figure 1*d*, white arrow). These data were compared with referenced spectra of the host rock and extant bone sample from a marine iguana (figure 3*a*,*b*,*e*,*f*). The Raman spectrum of the host rock clearly indicates calcium carbonate (figure 3*a*) as a typical phase for the environment in which fossils from the marine Middle Triassic are usually preserved [25]. Three analysed bone samples are characterized by typical bands assigned to the P-O and Ca-O modes (table 1). The extant iguana bone sample and non-altered bone spectra reveal a narrow band centred at, respectively, 959 and 960 cm^−1^, related to the symmetric P–O stretching ν_1_ (PO_4_)^3−^ modes of stoichiometric hydroxyapatite (HAp) [26–28,33,34] or carbonated apatites (CAp) [35,36] (figure 3*b*). These features are also sensitive to carbonate ($\text{C}{{\text{O}}_{3}}^{2-}$) and monohydrogen phosphate ($\text{HP}{{\text{O}}_{4}}^{2-}$) content [37] and should be considered in the context of an early stage of apatite mineralization [38]. A strong band with relatively low full width at half maximum (FWHM, approx. 15 cm^−1^) centred around 969 cm^−1^ is observed in the extant bone, which, due to fluoridation, is shifted towards higher wavenumber and is assigned to fluorapatite (FAp) [39]. The most intense band of the extant bone sample at 969 and 959 cm^−1^ is assigned to octacalcium phosphate (OCP) [38]. HAp is usually preceded by the formation of one or more calcium phosphate intermediate phases such as OCP and/or amorphous calcium phosphate (ACP) [38]. Examination of the fossil material (figure 3*c*,*d*) reveals two bands located at 969 and 940 cm^−1^ which result from, respectively, monophasic calcium phosphates or FAp [39], as well as the non-crystalline ACP or OAp [38]. The change in the ratio between HAp (CAp) and FAp in extant bone (figure 3*b*), as well as the non-altered fossil sample (figure 3*c*), is strongly linked to diagenetic processes and dissolution or transformation of the latter compound to the typically bone-forming phase. In the pathological plaque sample, the spectrum is dominated by a strong band located at 949 cm^−1^ and lower intense ones at 958 cm^−1^ and 920 cm^−1^ (figure 3*d*). The two lower bands originate from non-crystalline ACP or OAp [38] and the third one at 958 cm^−1^ is associated with residual non-altered HAp. The other bands located in the 450–400, 1080–1020 and 635–560 cm^−1^ regions originate from, respectively, the ν_2_ symmetric bending, ν_3_ asymmetric stretching and ν_4_ asymmetric bending modes of the (PO_4_)^3−^ groups [27]. Below 350 cm^−1^, Raman features are derived from translations of the O-Ca-O, O-P-O, OH^−^ ions, as well as liberations of (PO_4_)^3−^ ions. More detailed studies of extant and non-altered fossil bone spectra reveal bands in the 1590–1280 cm^−1^ region, as well as at approximately 870 cm^−1^, which, respectively are associated with organic compounds (e.g. amides; figure 3*b*, in the range of 1250–1750 cm^−1^) in extant samples [34,40] and amorphous organic carbon [41] in fossil samples. The occurrence of amorphous carbon might be associated with the very porous nature of the non-altered bone, creating a route for organic matter incorporation into the structure. The occurrence of amorphous carbon might be also associated with thermally altered intraosseous compounds, like proteins or lipids. Such bands are not observed in the spectrum of the strongly altered pathological plaque. Both extant bone and non-altered fossil bone are characterized by the presence of carbonate (CO_3_)^2−^ groups incorporated into the crystal structure of calcium phosphates (figure 3*b*,*c*). Bands located at 1093 and 878 cm^−1^ on the spectrum of modern bone sample represent (CO_3_)^2−^ anions partially occupying OH^−^ positions (A-type), while the band detected at 1078 cm^−1^ reflects partial (PO_4_)^3−^ substitution (B-type). Similar findings present in non-altered fossil bone confirm predominance of carbonated apatite in this type of bone (table 1) [27,29–31]. Such bands are not observed in the Raman spectrum of the pathological plaque sample, confirming its strong structural alteration. This is probably due to the removal of carbonate groups from the structure as a result of (micro)environmental processes, likely to be associated with bone formation under pathological conditions (see Discussion).

**Table 1.** Raman band assignment of extant, non-altered and pathologically altered bones.

Raman shift (cm^−1^)				
extant bone	non-altered cortical bone	pathologically altered bone (necrotic plaque)	band assignment	references
—	1188	—	ν_3_ asymmetric stretching mode of (PO_4_)^3−^	[26–28]
1093	1090	—	ν_4_ stretching mode of the (CO_3_)^2−^ (A-type)	[27,29–31]
1078	1070	—	ν_3_ bending mode of (CO_3_)^2−^(B-type)	[27,29–31]
1039	1034	1063, 1026	ν_3_ asymmetric stretching mode of (PO_4_)^3−^	[26–28]
969, 959, 924	969, 960, 940	958, 949, 920	ν_1_ symmetric stretching mode of (PO_4_)^3−^	[26–28]
878	870	—	ν_2_ bending mode of (CO_3_)^2−^	[27,29–31]
—	695	—	ν_4_ deformation bending mode of (CO_3_)^2−^	[32]
620, 591, 539	590	619, 582	ν_4_ bending mode of (PO_4_)^3−^and/or (HOPO_3_)^2−^	[26–28]
451, 432, 396	432	432	ν_2_ bending mode of (PO_4_)^3−^ and/or (HOPO_3_)^2−^	[26–28]
324	379	—	Ca-O-Ca bending mode	[26]
279, 234	282, 234	284, 265, 234	O-Ca-O bending mode	[26]

The low intense bands (3449, 3290 cm^−1^) on the reference infrared spectrum of host rock (calcium carbonate, figure 3*e*) and on non-altered fossil bone (3434, 3239 cm^−1^, figure 3*g*) are associated with water adsorbed on the surface or hydroxyl groups of the HAp structure. However, the low hydroxyl signal of non-altered fossil bone suggests a relatively low content of hydroxylated apatite with augmentation of the carbonate component of fluorine apatite's concentration. The extant bone sample (figure 3*f*) reveals a series of bands originating from methylene groups (3000–2800 cm^−1^), amide A and B (3400–3000 cm^−1^) and water molecules (3537, 3424 cm^−1^), similar to those previously linked to adsorbed water on the surface or a hydroxide of HAp structure. A few relatively strong bands with high values of FWHM centred at 3449, 3327, 3179 cm^−1^ in the pathological bone (figure 3*h*) are attributed to the stretching vibrational mode of molecular water arranged within the strongly modified structure of calcium phosphates. Here, the infrared spectrum indicates also four strong and narrow quantities (3694, 3665, 3619, 3594 cm^−1^) associated with the vibrational modes of OH^−^ groups as well as with the H-bonding pattern formed due to the interaction between proton and oxygen from phosphate units. In addition, a strong hydration might also generate a special type of molecular interaction (repulsive type of interaction) between the hydroxide units, which provides a shift towards higher wavenumber [42–44].

## 4. Discussion

### 4.1. Decompression syndrome of pistosaurs

The presence of decompression syndrome-related avascular necrosis in pistosaur limb bones and the occurrence of pistosaurid remains all around the globe in the Triassic times suggests that these animals were active swimmers. Although their remains are limited to near-shore sediments [4,6,8–10], it cannot be excluded that they were able to conquer open seas in the early Mesozoic. One of the strategies supporting global distribution of pistosaurs in open marine cold waters was more efficient metabolism, manifested by a high degree of vascularization of bone tissue [12] as in plesiosaurs. Even if they were not trans-oceanic long-duration swimmers, the water depths in near-shore and coastal environments were sufficient to allow decompression syndrome to develop. Diedrich [4,5] suggested that femoral shafts with thick and short shaft proportions, which end in irregular non-smooth joint surfaces, are typical for *Pistosaurus*. It appears that Diedrich actually describes the avascular necrosis condition of such bones manifested by subsidence of joint surfaces. Likewise, Sues [45] illustrated specimen SMF R.2011 in detail, but did not mention its abnormal appearance. An extensive epidemiologic study on avascular necrosis prevalence in European *Pistosaurus* is not possible, because pistosaur bones are extremely rare (Hans Hagdorn, personal communication) and beyond the scope of this study. Moreover, the necrotic plaque in SUT-MG/F/Tvert/43-1 occupies the subsidence area, confirmed by macroscopic and XMT observation.

### 4.2. Distinguishing septic and aseptic necrosis

The region of bone where septic AVN was found initially suggested decompression syndrome-associated aseptic necrosis. However, detailed examination of the surface of SUT-MG/F/Tvert/43-1, as well as macroscopic examination of specimens MHI 931 and NME 78.341, reveals periosteal reaction in a filigree-type pattern, characteristic of infection [46].

Owing to the fragmentary nature of pistosaur remains from Upper Silesia, it is not possible to study the opposing joint surface. X-ray microcomputed tomography revealed that the thin plaque at the articular surface is associated with bone tissue during life and not foreign matter fused to bone during fossilization. The presence of draining sinuses (figure 2*a*–*h*) and detailed XMT studies in three planes revealing a filigree or mesh-like pattern confirm that the thin plaque is pathologically modified bone tissue, not calcified cartilage, which is characterized by a more globular or sinusoidal pattern [47]. Moreover, no calcified cartilage was identified in the opposite side of the specimen SUT-MG/F/Tvert/43-1 and the split-like draining sinuses reach deep into subchondral bone (figure 2*d*,*e*,*g*).

Furthermore, the pathologically altered bone fragment is characterized by lack of carbonate bands (figure 3*d*) and strongly modified bone phosphate material, apparently caused by the pathological (infectious) alteration of bone tissue.

Septic arthritis is an infectious process of the synovium around joints, producing pressure, reducing or shutting off blood flow to the infected area, with resultant necrosis and consequent destruction of articular cartilage and erosion of the joints. It may be caused by several pathogens, most commonly bacterial (both suppurative and mycobacterial), rarely fungal. Septic arthritis has been so far described in extant crocodilians [46,48] and marine turtles [49,50], and more recently in a duck-billed dinosaur [20]. The studied pathologies (SUT-MG/F/Tvert/43-1 as well as NME 78.341 and MHI 931) are very similar to the infectious-associated destructive changes in zoological and anthropological materials [46,49,51].

### 4.3. Comparison of chemical composition of samples

The Raman spectrum of the pathological plaque differs from that of non-altered fossil and extant bones, with strong structural modification of initial phosphate apatite. The extant sample is dominated by fluoro-, carbonated- and hydroxyapatites, while non-altered fossil bone reveals predominance of the hydroxylated counterpart. The integral band intensities of the ν_1_ stretching region of (PO_4_)^3−^ and determination of the I_(960)_/I_(949)_+I_(979)_ ratio document an opportunity to estimate lower crystallinity rate of apatite of pathological plaque in relation to non-altered fossil and extant bone samples.

Increased band intensity at 949 cm^−1^ suggests increased structural vacancies in ACP or OAp due to the removal of carbonate groups and fluorine ions. The decrease of typical Ap band intensity and shift towards lower wavenumber and broadening (compare figure 3*a*–*c*) indicate that the variety of ions generating the homogeneous $\text{P}{{\text{O}}_{4}}^{3-}$ stretching environment observed in unaffected fossil bone has been replaced by a more heterogeneous one in pathological plaque. The pathological plaque sample lacks any signal typical for organic matter, which is present in the extant bone sample (amides and methylene groups) and non-altered fossil sample (amorphous carbon).

Infrared analysis of hydroxyl region (3800–2800 cm^−1^) showed predominant contribution of OH^−^ groups. In the non-altered fossil bone only a water signal, typically observed in fossilized bone apatite [52], was detected (figure 3*g*,*h*). Hence, higher incorporation of hydroxyl moieties into the apatite-like phase structures correlates with an increase of structural distortion in pathological bone. Oxygen metabolism has a significant role in the pathogenesis of arthritis [53,54] and reactive oxygen species (ROS) are relevant in degradation of cartilage [55]. Among ROS which are produced in infectious processes, the hydroxyl radicals, the neutral form of the hydroxide ion (OH^−^), are very common [56].

## 5. Conclusion

The results of our study show two different types of bone necrosis in the stem group of Sauropterygia and comprise the earliest record of septic bone necrosis in a fossil tetrapod, as well as the oldest case of bends-related avascular necrosis within the Sauropterygia clade. As with many other marine reptiles, *Pistosaurus longaevus* underwent dysbaric stress, with resultant avascular necrosis. Moreover, we conclude that these animals seem to be susceptible to joint infections, because of the diagnosis of septic arthritis in the three case studies presented herein. Furthermore, we demonstrated the apparent role of hydroxyl radicals in the pathophysiology of this ancient septic arthritis.

## Supplementary Material

Electronic Supplementary Materials

## Data accessibility

We declare that all data are included in the manuscript and electronic supplementary material.
