# Supplementary material for: Two types of bone necrosis in the Middle Triassic Pistosaurus longaevus bones: the results of integrated studies
Source: R Soc Open Sci. 2017 Jul 26;4(7):170204. doi: 10.1098/rsos.170204 (PMC5541542; doi:10.1098/rsos.170204)
Supplement: Electronic Supplementary Materials [file rsos170204supp1.pdf]

Two types of bone necrosis in the Middle Triassic *Pistosaurus longaevus* bones – the results of integrated studies

Dawid Surmik[1], Bruce M. Rothschild[2], Mateusz Dulski[3], Katarzyna Janiszewska[4]

Electronic Supplementary Material – supplementary figures S1-S4 with captions

[1]Park of Science & Human Evolution, 1 Maja 10, 46–040, Krasiejów, Poland and Faculty of Earth Science, University of Silesia, Będzińska 60, 41–200, Sosnowiec, Poland; dawid@surmik.pl. [2]Carnegie Museum, 4400 Forbes Ave, Pittsburgh, Pennsylvania, 15213 USA; West Virginia University School of Medicine, Morgantown, West Virginia 26506, USA; spondylair@gmail.com [3]Silesian Centre for Education and Interdisciplinary Research, 75 Pułku Piechoty 1A, 41–500, Chorzów, Poland, Institute of Material Science, University of Silesia, 75 Pułku Piechoty 1A, 41–500, Chorzów, Poland; mateusz.dulski@smcebi.edu.pl. [4]Institute of Paleobiology, Polish Academy of Sciences, Twarda 51/55, 00–818 Warsaw, Poland; k.janiszewska@twarda.pan.pl.

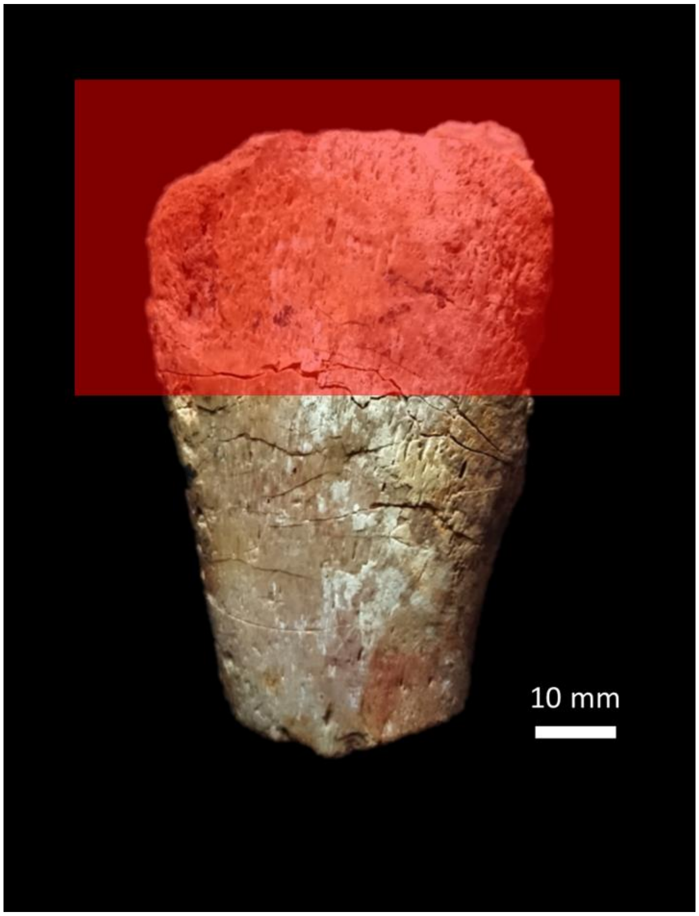

Supplementary Figure S1. The specimen SUT-MG/F/Tvert/43-1 in lateral view. The right area presents the part of specimen treated with acetic acid and covers all areas with which the samples to spectral studies were taken (compare figure 1 in main text).

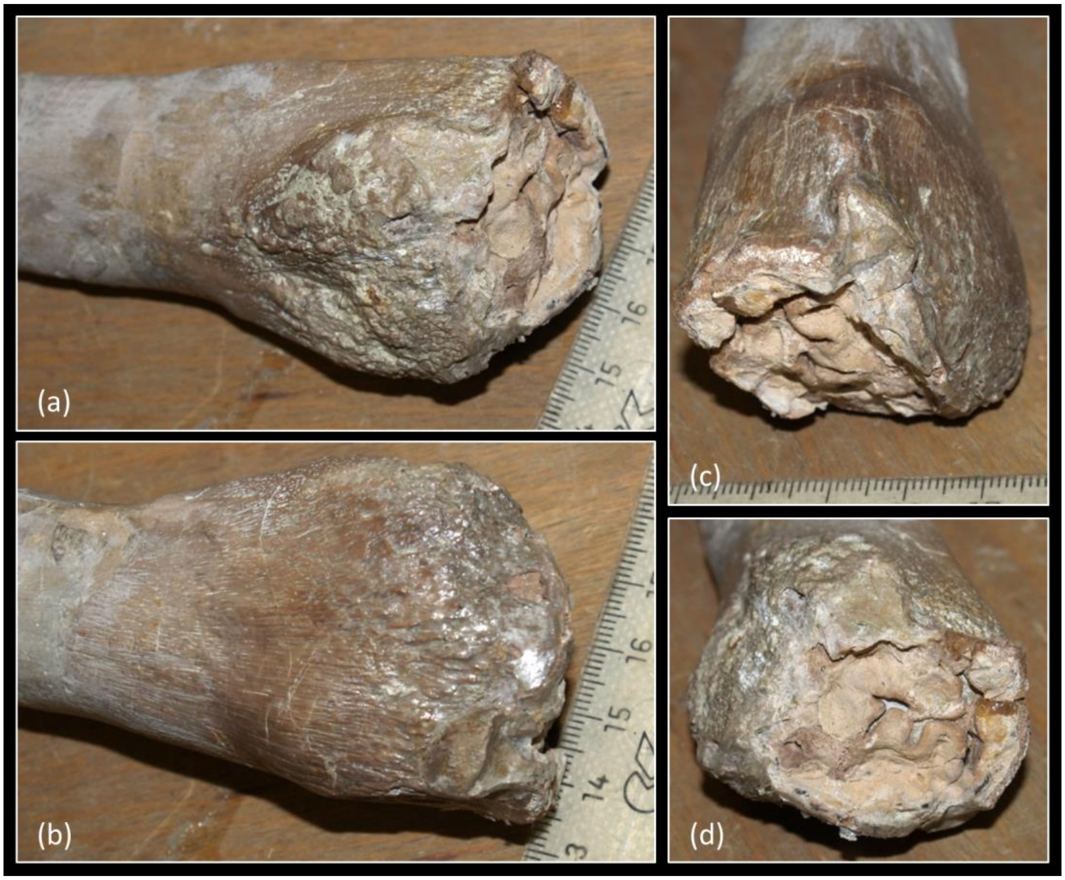

Supplementary Figure S2. The specimen NME 78.341 in various aspects. Courtesy Professor Siegfried Rein (Naturkundemuseum Erfurt).

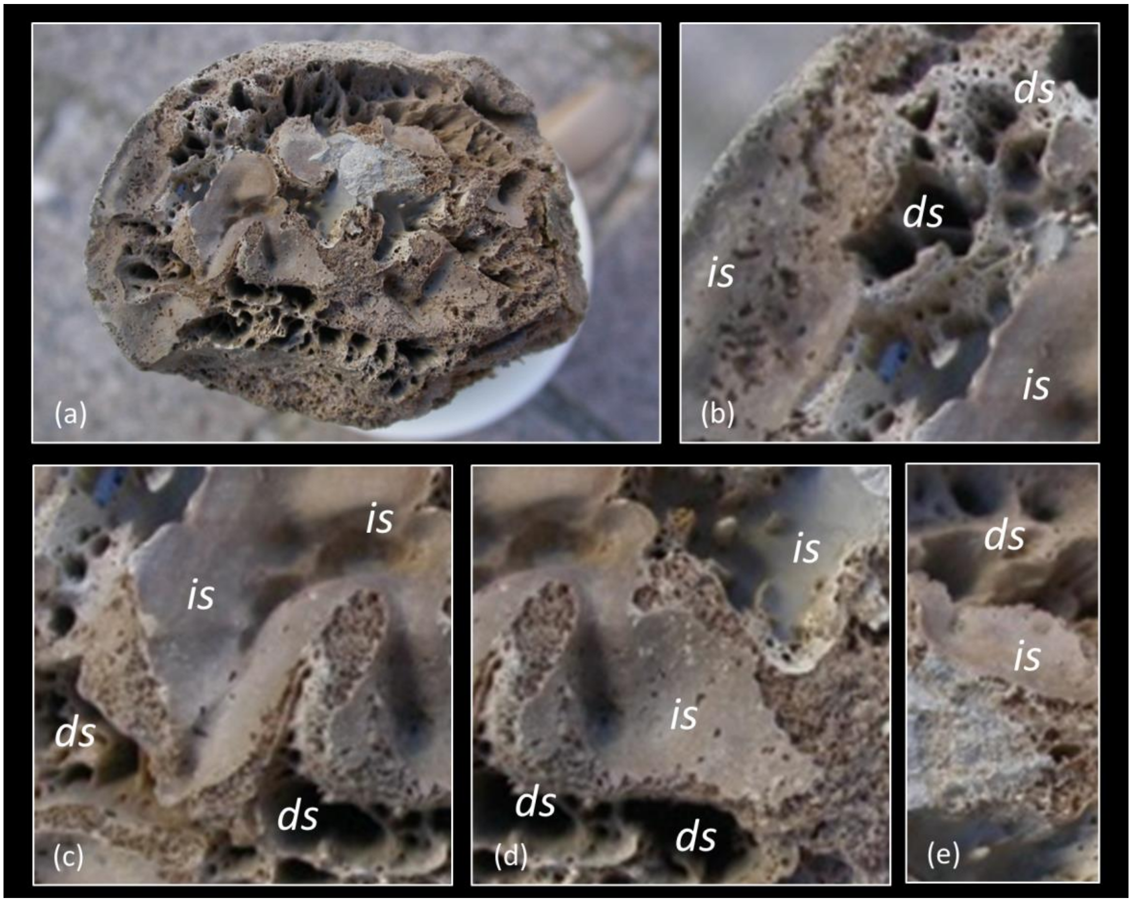

Supplementary Figure S3. The specimen MHI 931 in various aspects. A pathological plaque forms islets (*is*) interrupted with numerous draining sinuses (*ds*). Courtesy Dr. Hans Hagdorn (Muschelkalkmuseum Hagdorn Ingelfingen).

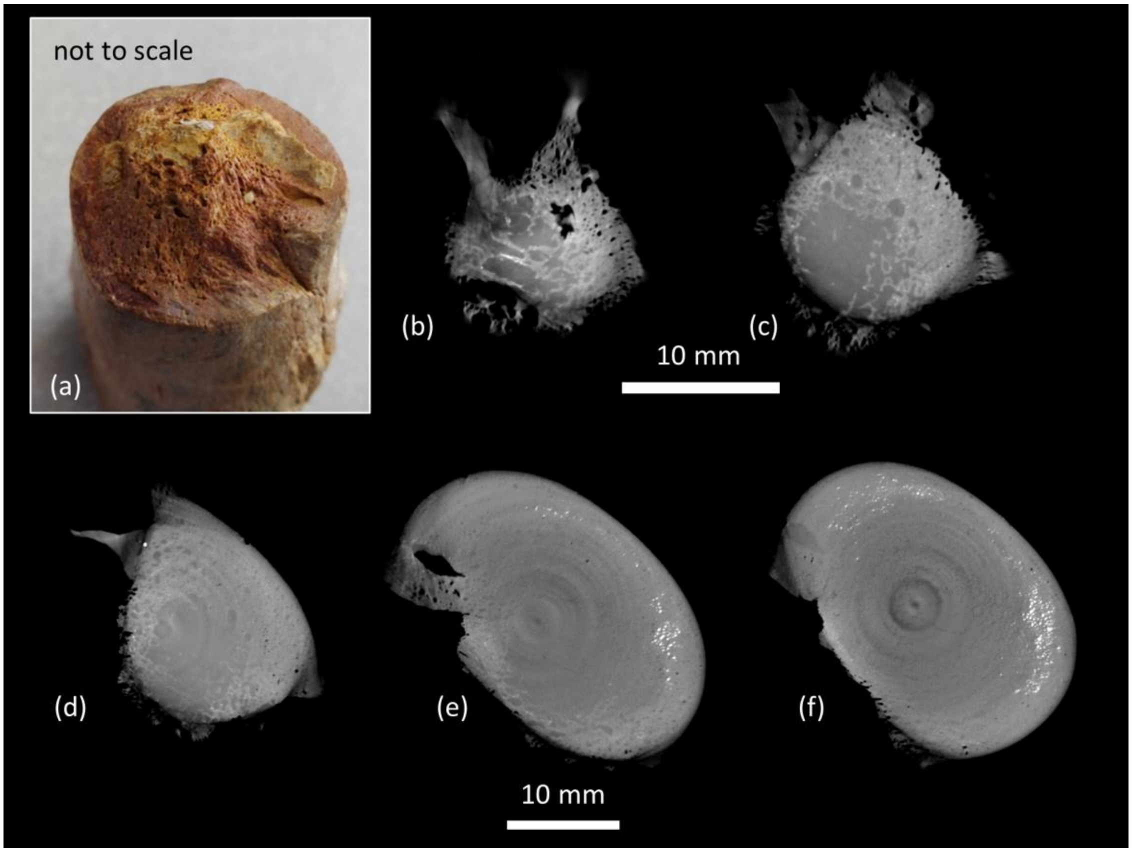

Supplementary Figure S4. The distal view of the specimen SUT-MG/F/Tvert/43-1 and several XMT sections showing no suspected calcified cartilage remnants within the bone tissue. The voxel size equals 39.95 µm.
